# Gut permeability in infants with cow’s milk allergy

**DOI:** 10.1186/2045-7022-5-S3-P155

**Published:** 2015-03-30

**Authors:** Svetlana Makarova, Leila Namasova-Baranova, Tatiana Borovik, Galina Yatsyk, Ivan Gmoshinskiy, Vladimir Mazo

**Affiliations:** 1 Scientific Center of Children's health RAMS, Moscow, Russia; 2 Institute of Nutrition RAMS, Moscow, Russia

## 

Our previous study revealed that newborns had a high level of absorption of human milk α-lactalbumin. [1] It decreased during the first month in the whole and non-allergic infants showed a 4- fold reduction of gut permeability at 25-30 days of life. But patients with early allergic symptoms have increased gut permeability in comparison to non-allergic infants. In this study we examined gut permeability in infants with food allergy.

29 infants with skin and gastrointestinal symptoms of food allergy and 15 non-atopic infants without any skin and gastrointestinal problems were under examination. Human milk α-lactalbumin was measured in blood serum by ELISA after breast milk feeding. Index of absorption was calculated according to the amount of consumed breast milk.

This study revealed high level of gut permeability in infants with skin and gastrointestinal manifestations of food allergy during the first year of life. Index of absorption of human α-lactalbumin in atopic patients was 4-fold higher than in control group at the age 3-6 months (p<0,01) and 5,5 fold higher than in control group at the age 6-12 months (p<0,01).

There was correlation between human α-lactalbumin absorption index and serum IgE level. High level of gut permeability is the purpose for using hydrolysed formula or amino acids based formula in patients with Cow's milk allergy.
